# A rapid assessment and response approach to review and enhance Advocacy, Communication and Social Mobilisation for Tuberculosis control in Odisha state, India

**DOI:** 10.1186/1471-2458-11-463

**Published:** 2011-06-10

**Authors:** Vishnu Vardhan Kamineni, Tahir Turk, Nevin Wilson, Srinath Satyanarayana, Lakbir Singh Chauhan

**Affiliations:** 1 International Union Against Tuberculosis and Lung Disease (The Union), The Union South-East Asia Office, New Delhi 110016, India; 2 Communication Partners International, 24 Dulwich Road, Springfield, NSW, 2250, Australia; 3 Central TB Division, Directorate General of Health Services, Ministry of Health and Family Welfare, Government of India

**Keywords:** tuberculosis, advocacy, communication, social mobilisation, rapid assessment and review

## Abstract

**Background:**

Tuberculosis remains a major public health problem in India with the country accounting for 1 in 5 of all TB cases reported globally. An advocacy, communication and social mobilisation project for Tuberculosis control was implemented and evaluated in Odisha state of India. The purpose of the study was to identify the impact of project interventions including the use of 'Interface NGOs' and involvement of community groups such as women's self-help groups, local government bodies, village health sanitation committees, and general health staff in promoting TB control efforts.

**Methods:**

The study utilized a rapid assessment and response (RAR) methodology. The approach combined both qualitative field work approaches, including semi-structured interviews and focus group discussions with empirical data collection and desk research.

**Results:**

Results revealed that a combination of factors including the involvement of Interface NGOs, coupled with increased training and engagement of front line health workers and community groups, and dissemination of community based resources, contributed to improved awareness and knowledge about TB in the targeted districts. Project activities also contributed towards improving health worker and community effectiveness to raise the TB agenda, and improved TB literacy and treatment adherence. Engagement of successfully treated patients also assisted in reducing community stigma and discrimination.

**Conclusion:**

The expanded use of advocacy, communication and social mobilisation activities in TB control has resulted in a number of benefits. These include bridging pre-existing gaps between the health system and the community through support and coordination of general health services stakeholders, NGOs and the community. The strategic use of 'tailored messages' to address specific TB problems in low performing areas also led to more positive behavioural outcomes and improved efficiencies in service delivery. Implications for future studies are that a comprehensive and well planned range of ACSM activities can enhance TB knowledge, attitudes and behaviours while also mobilising specific community groups to build community efficacy to combat TB. The use of rapid assessments combined with other complementary evaluation approaches can be effective when reviewing the impact of TB advocacy, communication and social mobilisation activities.

## Background

Tuberculosis remains a major public health problem in India with 1.98 million incident cases in 2009 accounting for 1/5^th ^of all TB cases reported globally [1,2]. The Revised National Tuberculosis Control Programme (RNTCP) operating on the principles of Directly Observed Therapy - Short course (DOTS) has been operational for over 12 years, initiating more than 11 million patients on treatment, and saving an additional 2 million lives [2]. RNTCP has sustained new sputum positive (NSP) case detection rates of over 70% and treatment success rate over 85% nationally since 2007, in line with global targets for TB control [2]. TB mortality and prevalence in the country has witnessed a reduction by 43% and 67% respectively compared with 1990 figures, indicating progress towards achieving TB related targets of the United Nations Millennium Development Goals [1,2].

However, lack of public awareness coupled with limited involvement and engagement of communities and non-governmental organisations (NGOs) have been identified as challenges impeding progress toward TB control. The World Health Organisation (WHO) has identified the need for innovative approaches to engaging diverse private and public providers to widen the network and improve accessibility of services [3]. WHO recommends an Advocacy, Communication and Social Mobilisation (ACSM) framework for national TB programmes that aims to address 4 key challenges; case detection and treatment adherence, stigma and discrimination, empowering affected people, and lastly and perhaps most importantly, mobilising political commitment and resources necessary for TB control [3]. As such, ACSM activities are seen as an important and necessary step in eliciting greater awareness and engagement in TB control in order to achieve global TB targets [4].

### Project Setting

In October 2007, on the recommendation of the India Country Coordinating Mechanism, the International Union Against Tuberculosis and Lung Disease (The Union) was identified as a sub-recipient to the Central TB Division (CTD)-Ministry of Health and Family Welfare, to collaborate in supporting implementation of its ACSM component in Odisha state and provide technical assistance to the state under Global Fund for AIDS TB and Malaria Round 4, Phase II funding. Odisha state has a population of 36.8 million in 31 reporting districts, 314 blocks and 51,350 villages with a population density of 236 people per sq.km. Odisha has been identified as the poorest state in India with an estimated 39.9% of the population living below the poverty line [5]. RNTCP was launched in Odisha state in 1997 with support from the Danish International Development Agency (DANIDA). The programme has witnessed a phased expansion covering 14 predominantly tribal districts during phase I (Dec 1996-Mar 2003). The programme was later expanded to cover the remaining 17 districts in the state during a second phase ending March 2004. An Odisha state specific ARTI (Annual risk of TB infection) study conducted in 2003 revealed that the ARTI was 1.7% for the state, which is higher than the national average of 1.5% [6].

As a first step to understanding prevalent sub-national ACSM planning and programming, and to evaluate existing ACSM practices, The Union conducted a comprehensive ACSM review of RNTCP in Odisha State. While identifying programme strengths, the review mission observed reduced interest in approaches such as Advocacy, Communication and Social Mobilisation among health sector stakeholders [7].

As a follow-up to the ACSM review, a state specific ACSM strategy was developed by The Union in collaboration with state stakeholders and endorsed for action by the Odisha RNTCP. A cornerstone of the ACSM strategic document was the recognition of the need to enhance community ownership, involvement and participation to drive TB control activities, with inclusive consultations advised with all TB stakeholders and targeted beneficiaries in TB control planning and development. In order to achieve this end, the strategy recommendations were that Odisha RNTCP strengthens its ACSM public private partnerships to involve all key stakeholders/organisations with competencies in core programme delivery areas [8]. The strategy also recommended the implementation of an integrated, multi-level, ACSM plan encompassing community based, interpersonal communication activities addressing a broad range of programme areas and targeted interventions for the most vulnerable groups.

In alignment with the Odisha ACSM strategy, The Union, in consultation with the Directorate of Health Services, Odisha state, developed a Project work plan addressing aspects of the ACSM strategy to support Odisha RNTCP in low performing districts. Specific objectives for the ACSM Project as defined in the proposal document included, facilitation for the increased adoption of DOTS by all health care providers in Odisha, increased ownership of DOTS by civil society including Panchayati Raj Institutions (local self-governing institutions), community based organisations, Women's Self Help Groups, and lastly the facilitation of increased accessibility to DOTS services in tribal and coastal areas in the state [9]. To meet stated objectives, activities proposed under the Union project focused on general as well as targeted interventions.

Implementation of social mobilisation activities was initiated in April 2008, and continued intensively over 21 months until December 2009. The last quarter of Project (Jan-Mar 2010) involved phasing-out activities, project documentation and end-term evaluation.

### Project conceptual framework and service delivery implementation model

The project concept identified community-based institutions to implement project activities for TB control. Working in close coordination with RNTCP, these institutions designated as 'Interface NGO's (IFNGOs) were tasked with delivering all social mobilisation interventions aimed at promoting community action for TB control. The role of IFNGOs while translating RNTCP policy into action, was geared to bridging gaps and act as an 'interface' between community and public sector stakeholders (RNTCP).

**Figure 1:**
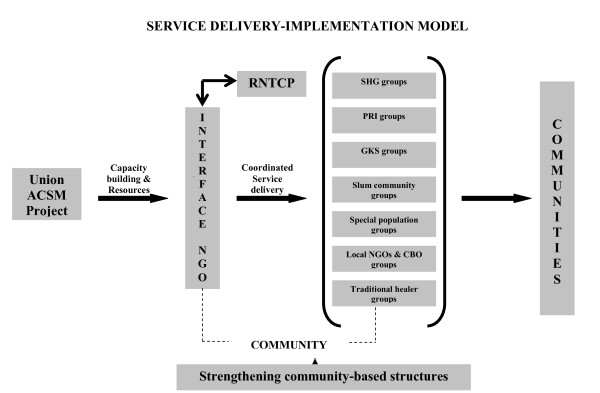
**Service delivery - Implementation model**.

Project support of IFNGOs was seen as critical and included technical skills transfer on basic concepts of TB, material and financial resources to implement ACSM activities. Sensitisation activities involving community groups adopted specific tailored messages for specific audience segments in line with health communication objectives [10]. Messages were designed to improve suspect referrals for TB diagnosis in areas with low case detection, emphasizing the need for treatment completion/adherence in areas with poor treatment outcomes for TB, improve awareness of TB and the availability of free TB services at public sector facilities and lastly, reducing stigma and discrimination. A diagrammatic representation of the conceptual framework provided in Figure 1 illustrates project support through training, capacity building and provision of financial and material resources to IFNGOs. The scale of ACSM activities, in terms of numbers organized and participants reached is illustrated in Tables 1 and 2.

**Table 1:** State level ACSM activities for TB control

	*Activity*	*# organized*	*# Participants*	*Districts*
1	NGO Workshop	2	58	17

2	IMA Workshop	1	37	19

3	ESI Workshop	1	15	4 zonal offices

4	Zonal Taskforce Meeting	1	20	6 Medical Colleges

5	CF Training	1	6	All

6	STS Training	4	105	All

7	ACSM Workshop for IEC Personnel	1	22	-

8	ACSM Taskforce Meeting	2	27	-

9	Communication Material Development Workshop	2	15	-

10	Patient Association Meeting	3	79	31

11	Corporate Sector Involvement Workshop	1	27	10 districts

12	World TB Day Observation	3	345	-

13	State Tribal Strategy Document - RASTA O	1	-	2 sample districts

14	Tribal strategy plan for Gajapati district	1	-	1

15	KAP study	1	-	12 sample districts

**Table 2:** District level ACSM activities for TB control

	*Activity*	*# organized*	*# Participants*	*Districts*
1	SHG member sensitisation	4305	148326	16

2	Slum community sensitisation	560	12706	5

3	Special populations group (fisher folk & stone quarry, Tibetan community etc.)	356	12070	4

4	PHC staff sensitisation	172	8114	16

5	BEE, BPO sensitisation workshop	28	757	15

6	NGO sensitisation workshop	25	595	25

7	PRI members (local self-governing institutions) sensitisation	485	9383	16

8	Local NGO and CBO sensitisation	557	18660	16

9	GKS member sensitisation	993	12528	9

10	Traditional healer sensitisation	100	2689	5

11	Patient association meeting	32	897	12

12	Exhibition in weekly hat	8	85000	6

13	Interactive stall on festive occasion	29	8026	6

14	Monthly meeting-IF NGOs and district functionaries	352	2318	20

15	ASHA training	155	3854	13

16	AYUSH doctor training	28	636	18

17	Private practitioner sensitisation	8	162	6

18	ESI staff sensitisation	1	31	1

19	Corporate sector staff sensitisation	9	65	5

20	Kalyani club sensitisation	8	297	5

### Survey Objectives

The objectives of the survey were to assess strengths, advantages/disadvantages of Project specific ACSM approaches and processes at state and district level in Odisha. This included Project performance in implementing both general and special ACSM intervention activities. A second survey objective was to assess the extent to which the long term, specific and operational objectives of the Project had been achieved. This involved documenting comparisons between the Project objectives and Project targets and action plans as compared to actual accomplishments in the period under evaluation. Lastly, the survey was designed to assess and document the sources, nature, level, uses and impact of the ACSM strategy and Tribal strategy to the Project and consequently the state RNTCP and provide recommendations. The importance of this survey assessment was founded on the proposition of the need for ongoing Project reviews at strategic periods to identify performance targets, and the adoption of a culture of quality assurance and continuous improvement in ACSM approaches and practices.

## Methods

It was acknowledged that a combination of qualitative and quantitative methods would be most likely to provide the most reliable and valid research results with the approach supported by a number of other authors [11,12]. As such, a combination of approaches for the Odisha ACSM Project evaluation was considered most appropriate. The evaluation factored information from the following primary and secondary sources: field work, data-analysis, literature review, qualitative and quantitative assessments, and empirical findings from the project.

### Stage 1 - Rapid assessment and response

Rapid assessment and response (RAR) methods have been successfully applied to many public health issues in low and middle income countries [13,14]. The approach is seen as a cost-effective, pragmatic method of public-health research, primarily used in developing and transitional countries [15,16]. The rapid assessment method used for the end-of-term Project evaluation comprised elicitation research with a number of key-informants. Additionally, focus group discussions were conducted with a range of programme beneficiaries including general and patient populations.

Three field teams were identified, comprising of 4-6 specialists representing GFATM, WHO, The Union, and RNTCP staff and consultants. Each field team was allocated a field visit plan to cover in all a total of 9 districts across the State (total districts in state 31). Standardized assessment tools and discussion agenda were developed through discussion by all team members at a review planning workshop, prior to field deployment. Instruments developed consisted of a number of semi-structured questionnaires with specific questions aimed at the different stakeholders groups including discussion agenda for key informants (RNTCP general health providers), community groups (Self-Help Groups, Local Self-Governing Institutions and Village Health and Sanitation Committees), interface NGOs as well as target groups of programme beneficiaries including TB patients, vulnerable groups (slum, coastal and tribal communities) and general population groups.

The assessment tools were designed to elicit responses to a number of Project key performance indicators. Field visit locations were decided upon in conjunction with the Directorate of Health in order to include consultations with key informants from RNTCP, and visits to a range of health facilities and communities in 9 districts (2 urban, 2 coastal, 2 rural, 2 tribal and 1 urban/rural district). Additionally, where time permitted, field teams were provided with discretionary authority to deviate from the arranged locations in order to conduct spontaneous visits to other health facilities or district locations to minimize potential bias in conducting discussions only with sensitized groups. The three teams of approximately 4 reviewers each comprising international, and national technical advisors were supported by district teams of another 3-4 local support staff (6-8 field staff in total for each team) to facilitate meetings, conduct translating duties and provide insights into the project roll-out. The method provided each review team with opportunities to consult with a wide range of stakeholders and beneficiaries across geographically diverse areas of Odisha state. The rapid assessment fieldwork period lasted approximately 4 days.

Facilitated discussions and semi-structured interviews were conducted with a number of key informants including Chief District Medical Officers, District TB Officers, representative from the India Medical Association, Community Based Organisations, Self Help Groups, and local self-governing institutions. Other district level functionaries interviewed included Mass Education and Information Officers, District Programme Managers, Senior Treatment Supervisors, Block Extension Educators, Block Programme Officers, Medical Officers - District TB Centre, Laboratory Technicians, Multipurpose Health Supervisors, Multipurpose Health Workers and DOTS Providers.

A number of other front-line health workers were also interviewed including Accredited Social Health Activists (ASHA), Anganwadi Workers (AWW), Community DOTS Providers, AYUSH (Alternative medicine) Doctors, Traditional Healers, Project Interface NGOs (IFNGO) as well as other NGOs. Interviews and discussions were also planned with programme beneficiaries and other target groups including Category 1, Category 2 and MDR patients under treatment, treatment defaulters, as well as successfully treated patients. Patient case studies were recorded in all districts to identify any significant themes and emerging patterns of behaviour. Wherever possible, general target populations were also interviewed in a range of social settings to identify general community health priorities, and TB knowledge, attitudes and behaviours (KAB).

The elicitation approach was designed to initially identify recall of specific Project activities to distinguish their potential impact from the range of other RNTCP and donor funded activities. Respondents who recalled Project activities were further probed on the knowledge gained following exposure to the interventions and the range of specific messages they recalled. The elicitation approach included further probing to identify if the information received through the activities was new information or simply provided a refresher to previous learning's. Researchers were also asked to probe for any perceptions in changes in attitudes and behavioural intentions resulting from the Project activities and finally, what respondents did as a result of these interventions.

During the field visits, notes were compiled by both the moderators and other field staff during each discussion session. The notes were compiled in a question-by-question format according to the assessment tools, to capture what the individual or group respondents had to say regarding each theme. The on-site summaries were enhanced through dialogue between team members immediately following the elicitation sessions, to clarify any key themes and issues, or potentially ambiguous responses. Transcripts of responses of the discussions with program beneficiaries, and key informants, were recorded by hand in note-books, comprising of approximately 40, A4 pages.

Field reports identified that around 223 stakeholders from the health sector, including state and district level IEC/ACSM staff, were consulted on various aspects of the programme. Discussions were also conducted with approximately 196 programme beneficiaries including TB patients, slum communities and tribal populations. Case studies were also recorded from a number of TB patients and DOT providers. Additionally, photographic records of the field-trips were also collected by each team to identify key issues sighted during the evaluation.

### Stage 2 - Literature review

Prior to and following the fieldwork, desk research was conducted of a range of literature to supplement the field work data. A number of operational and action plans provided by the project were reviewed, as well as reports on ACSM and needs assessment mission [7], ACSM strategy document [8], Tribal strategy [9], and the State Knowledge, Attitudes and Practices (KAP) survey [17]. Additionally, literature on psychological and behavioural change theories was also reviewed in relation to the Project approaches and activities identified during the field visits. This provided a comprehensive overview of findings, as well as empirical data on Project planning, implementation and evaluation. This data was then coupled with qualitative findings and observations compiled through the in-field study.

### Stage 3 - Field work analysis

Analysis of all the teams data was carried out in two iterative stages in order to build theory from the case study research, within-group (in the case of group discussions) and from individual responses (in the case of semi-structured interviews-SSIs) to explore cross-case patterns [18]. The content analysis was seen as a process of identifying, coding and categorising of the primary patterns contained in the data. Much of the knowledge on these approaches has emanated from grounded theory where *constant comparative methods *are used to describe the process of defining and separating themes by continuous reading of the raw data [19]. Grounded theory provided a systematic way of examining the qualitative data from the perspective of those who were actually experiencing the phenomena [20]. These combined approaches were designed to more accurately identify the extent of the Union Project interventions as well as identify what other factors may have played on community and individual TB knowledge, attitudes and behaviours.

First, within-case analysis involved writing up and summarizing key issues emanating from within the stakeholders and beneficiary discussions. The second step involved individually analysing responses from the various cases to identify a range of behavioural predictors including changes in TB knowledge and attitudes as a result of the interventions, any perceived changes in social norms of health providers and beneficiary groups, and perceived behavioural control (community efficacy and self-efficacy) to make the required health protective behavioural changes [20]. Summary notes on the cases were then compiled, followed by cross-case analysis. The same approach was adopted with both health providers and programme beneficiaries, following which KAB comparisons across these two predominant groups were conducted. The analysis ensured that a more holistic and contextualized understanding evolved of the key themes explored. These analyses were in-turn, cross referenced against available secondary data sources and other empirical findings. Following analysis of field notes and case studies a general consensus was reached by team members on the key themes and amalgamated issues.

### Stage 4 - Verifiable indicators

Discussions were also conducted with Project staff in the initial days of the evaluation mission to identify methods to verify that activity outputs and other process indicators set out in the activity plans had been achieved. Verification of workshops organised by the Project was achieved by randomizing a selection of each 7^th ^workshop conducted in a number of Project districts and requesting copies of the attendance lists from the Project central office. Verification of production and dissemination of publications resources was achieved by requesting copies of the delivery dockets of a number of items of printed materials in project activity areas. Additionally, qualitative indicators were assessed through discussions on recall of sensitisation and training workshops for a wide range of stakeholder groups, feedback on IEC materials received, and sighting of the printed materials.

## Results

Seven key themes emerged from the synthesis of Qualitative data. Key themes from the assessment included, (i) individual and community advocacy for TB, (ii) community engagement and social mobilisation, (iii) knowledge, attitudes, and beliefs about TB and treatment literacy (iv) systems and facilities for TB treatment and care (v) stigma and discriminating attitudes, (vi) treatment and adherence behaviours and (vii) health worker, family and community support. Interpretation of these themes across the evaluated intervention districts identified how a number of major factors may have interacted to affect TB KAB including structural, socio-economic, and cultural factors, as well as the social context, including community settings, the roles of stigma, caste and gender issues, geographic locations, and quality of service delivery. The following are the evaluation key qualitative findings in relation to a number of themes identified.

### Stakeholder findings

Evaluation findings following interactions with RNTCP, general health staff providers revealed that the Project had significant positive impact in strengthening the Odisha RNTCP programme. Capacity building initiatives for different categories of general health staff were typically appreciated and as a result provided increased motivation to engage with other stakeholders in TB control. This was identified with some minor exceptions, through an improved level of cooperation between RNTCP and interface NGOs. Evaluation teams observed that perceived awareness about RNTCP and the DOTS strategy had improved over the last 2 years in the project areas. Discussions with a number of CBOs/NGOs regarding TB ACSM activities and support issues also demonstrated good capacity and motivation to conduct these specific interventions.

Traditional healers were found to be more involved in TB case detection through suspect referral as a result of the Project sensitisation workshops. These informal providers in tribal areas were seen by a number of community respondents as often more trusted sources of health information. Traditional healers and AYUSH (alternative) medical practitioners reported that they were also more knowledgeable of the symptomatology of TB and the mode of transmission of the disease following the Project sensitisation workshops. These traditional health providers reported referring suspects for microscopy that was validated by the evaluation team through recordings in laboratory registers in selected tribal districts. Their involvement and improved ability to counsel suspects about TB and advocate DOTS at the community level was also observed during the evaluation.

Improved coordination between Block Extension Educators (BEE) and Block Programme Officers (BPO) under the National Rural Health Mission (NRHM) was also identified through consultations. Also noted was the increased involvement and coordination of general health care staff following sensitisation workshops. As a likely result of capacity building and engagement of front-line field staff and other health providers, a number of improvements in TB control outcome indicators were observed by the teams, especially in relation to referrals and case finding. Feedback from health workers also confirmed that demand for DOTS by the public had increased as a likely result of improved mobilisation of these front line health workers.

Evaluation findings based on in-depth interactions with district level stakeholders suggested that Project supported interventions were providing considerable support for increased involvement of civil society organisations. Interventions were acknowledged to have been planned in consultation with district stakeholders. Sensitisation of Senior Treatment Supervisors (STS) was found to have improved their confidence and skills in interpersonal communication (IPC). These IPC skills were not identified as part of these staff's original modular trainings. The observation of increased involvement of STSs in the implementation of ACSM related activities was also noted by public health staff. Review teams reports also identified increased involvement by Block Programme Officer's and Medical Officer's-in-charge during RNTCP monthly and sector review meetings. Communication materials provided by the Project were also appreciated by staff attending workshops and were seen to have helped in their education and advocacy efforts.

Findings from CBOs and NGOs also showed awareness of an increased number of social mobilisation and sensitisation activities conducted as a result of the Project. Evaluation findings showed a perceived, enhanced confidence and greater efficacy by these civil society organisations to conduct TB control activities. Also noted was an increased engagement in RNTCP NGO schemes (30 NGOs applied for the ACSM scheme in the one visited district alone). IFNGO staff also reported adequate IEC materials were made available by the project at each meeting, and that the coordination with various levels of health staff was supportive at all levels. This led to increased cooperation in the comprehensive roll-out of the ACSM strategy. While formal memoranda of understanding and collaboration between NGOs and RNTCP for all schemes have witnessed a 28% improvement during the project period, a 20% improvement was seen more specifically for ACSM collaborative schemes (Table 3). It is worth noting the project period coincided with the release of new RNTCP - NGO collaboration schemes.

**Table 3:** RNTCP-NGO collaboration

FORMAL MOU for RNTCP-NGO collaboration (Baseline-b and Project End Term-e)			
	Indicator	# NGOs	Period
1	No of NGOs involved in Odisha state in all RNTCP schemes	**57**	3^rd ^Q 2007-b
2	No of NGOs involved in Odisha state in Health education and Community outreach (ACSM)	**29**	3^rd ^Q 2007-b
3	No of NGOs involved in Odisha state in all RNTCP scheme	**73**	4^th ^Q 2009-e
4	No of NGOs involved in Odisha state in ACSM-RNTCP scheme	**35**	4^th ^Q 2009-e

Feedback from front-line health workers such as ASHA and Anganwadi Workers (AWW) also showed their increased engagement in TB control activities as a result of trainings. A number of ASHAs and AWWs reported they were now aware of the signs and symptoms of TB, and of the new referral guidelines following sensitisation by the Union project. Also reported by some ASHAs was an increased confidence drawn from new knowledge in identifying chest symptomatic cases. The training module publication was also reported as being a very handy reference to build competence and confidence to deliver ACSM activities to communities.

Treatment literacy was also seen to have improved with front-line health workers reporting greater confidence in getting support at the designated microscopy center when they accompanied patients, as well as self-confidence to handle TB cases in the community. A number mentioned that they were now actively advocating DOTS during meetings with community groups and sensitizing local self-governing institutions. A ASHAs counselling booklet was also seen as a useful tool which helped in counselling patients/suspects and members of the public. In general, findings showed that frontline health workers were now more engaged in TB control than was identified in the 2008 ACSM review, although the focus of their activities was still seen largely on the provision of DOTS. However, there were some persistent complaints, as per the initial 2008 ACSM review, by ASHAs and AWWs that their incentive payments for successful treatment completion were not paid on time. This resulted in de-motivation of a number of these community volunteers.

Consistent with findings from other health worker groups, sensitisation and training workshops were found to have also re-energized interest in TB control within community groups such as SHG, PRI, GKS groups. It was noted that a number of these groups employed front-line health workers such as ASHAs/AWWs on their committees, with some community groups also including successfully treated patients. As a result feedback from the groups demonstrated greater competence to engage in suspect identification, as well as counsel suspects about TB. Community and individual discussions on TB reported by committee members also identified increased advocacy for DOTS at the community level. Their perceptions were that now communities were more aware of TB symptoms and the location of health facilities, as well as having a greater awareness and social ownership of TB.

Although there was only one State level patient's association identified in the capital -Bhubaneswar, it was noted that in rural areas there was some degree of assimilation of former TB patients into community groups. This had the effect of increasing the confidence of these patients to advocate for TB control, and also engage in suspect identification and referral for diagnosis. In communities where TB patients had been integrated into these community groups, stigmatizing attitudes were generally perceived to be reduced and a number of suspects had been referred for treatment directly by the cured patient. Although, issues of women deferring treatment as a result of TB stigma were still identified, the greater involvement of successfully treated TB patients in these community groups increased the profile of TB generally, and also helped to reduce stigma and promote treatment adherence, thus reducing the chances of treatment delay.

Sensitisation and training workshops were seen to have increased engagement in case detection within these communities with a number of specific examples identified, most particularly, in rural and remote settings. Referral slips used by SHG, PRI were also being acknowledged by the system. A number of examples of increased networking and motivation were also noted among these stakeholders.

The involvement of Interface NGOs for ACSM implementation was seen as an innovative approach to addressing specific issues within problem areas. It was observed that training and engagement of IFNGOs had enhanced their capacity to address TB control, with these agencies now filling an important gap previously not addressed. Although there were some pockets of suboptimal trust and cooperation noted, this was understandable given the previous suboptimal to non-engagement of NGOs in Odisha into TB control activities, and a common resistance to change within the public sector. This resistance was observed to have diminished in areas where NGOs and RNTCP staff had developed positive relationships and were working cooperatively.

A number of IFNGOs were very responsive in fulfilling obligations under the Project, and were seen to be actively involved in suspect identification, and referral and tracing of defaulters and patients who were irregular in their treatment. The IFNGO staff noted their satisfaction with the capacity building activities of the Union Project as well as other capacity building opportunities afforded by the State. Staff opined that the ACSM activities had had a definite impact on the awareness of TB among the general public and as a result referral of suspects had also increased. IFNGOs had also taken a lead in the formation of district level patient's associations which were working to build TB awareness and reduce stigmatizing attitudes, particularly toward females. IFNGO staffs were also familiar with the Patient TB charter and identified more active advocacy for patients' rights. However, it was noted that this process may have required the charter to be popularized.

### Project beneficiaries' findings

Findings from Project beneficiaries also indicated an increased awareness of TB care and other services provided by former patients and other community members. This included improved knowledge on TB signs and symptoms. Although, attitudes towards public sector services were seen to be improving in remote areas, the private sector was still seen as a trusted provider.

A perceived increase in the awareness about TB care and support services was also noted in urban areas through patient case studies. Patients interviewed in a number of clinics were aware of the symptoms of the disease and understood its transmission, duration of treatment, sputum follow up schedule, and were confident that DOTS provided an effective cure. A number of patients also reported that they referred other symptomatic persons for sputum examination demonstrating good social mobilisation of a number of these patients.

General populations also demonstrated confidence about the location of TB services and their nearest health facilities although discussions in a remote village community identified the difficulties of locating these services in distant towns by tribal populations. Teams also reported increased knowledge about ASHAs, community providers and community DOT provision, as well as increased knowledge about duration of treatment and the need for follow up sputum examinations. These knowledge indicators were not as evident in remote populations where patient delay, as a result of the need to work, and the travel and other associated costs of health seeking were found to be significant barriers to early treatment seeking among the poor and tribal populations. The pre-treatment, and during treatment counseling provided was also found to be suboptimal in tribal areas. Examination of records and reports revealed that case holding also continued to be a problem among tribal and migratory populations as a result of geographic and economic barriers.

### Empirical findings

Empirical findings revealed that a total of 8236 ACSM activities were conducted at state and district level by the project that included training and sensitisation of health care providers, capacity building activities, engagement of NGOs, and the development of community based resource materials and toolkits. Odisha RNTCP addressed aspects of the ACSM strategy such as advocacy initiatives and authoritative reinforcement of RNTCP policy through senior public health officials, which were beyond the scope of The Union Project interventions.

The Project implemented general population based ACSM approaches during year 1 with a focus on low performing districts as identified by the state. However, the second year of implementation focused both on general population based approaches, as well as targeted interventions in selected districts so as to contribute towards improving TB control in Odisha state. One of the Project anticipated case findings outcomes conceived at proposal stage was an additional 10,000 TB cases notified in Odisha state over a project period of 3 years.

In terms of suspect referrals and case notification, the baseline year of 2007 was considered for case notification and for the project outcome assessment. Case notification during the project implementation period 2008 and 2009 was compared with the baseline year. All quarterly reports for the period 1Q 2004 until 4Q 2009 were assessed to analyze trends on suspect referral and total TB cases. The analysis revealed an overall additional gain of 2860 TB cases and 14873 suspects in Odisha State during the project period 2008 and 2009 (Table 4 and 5). An increasing trend was also identified for cases notified and suspect referral during the period 2004-2009. The increasing trend observed in suspect referral and case notification may be attributed to an improvement in RNTCP programme performance in Odisha State. This improvement may be due to diverse factors such as improved RNTCP programme service delivery, general health services ensuring availability of TB services, enhanced supervision and monitoring mechanisms, as well as improved awareness among the community to seek early care and knowledge regarding the availability of TB services.

**Table 4:** Additional gain in Total TB cases 2008-2009 (Implementation period)

1	Baseline year-2007	Total cases - 49285
2	Increase in 2008 over baseline	1746 (Total cases in 2008 - 51031)
3	Cumulative increase in 2009	2860 (Total cases in 2009- 52145)
4	Overall increase observed in Project period:	2860

**Table 5:** Additional gain in Total TB suspects 2008-2009 (Implementation period)

1	Increase in 2008 over baseline **Y **2007	9652
2	Cumulative increase in 2009	14873
3	Overall increase observed in Project period	14873

While project specific ACSM activities may have contributed to this increase during the implementation period, the precise measure of that contribution is more tenuous i.e., isolating project activities to the overall impact (improved case notification), given the nature of predominantly used general population based approaches. However, as monitoring and evaluation (M&E) mechanisms improve, and targeted interventions gain traction it is believed that a substantial impact of improved case notification as a consequence of ACSM specific targeted intervention approaches may be accurately measured over the longer duration (3-5 year intervention period).

Treatment outcomes for 15 low performing districts during 4Q 2007 were compared with end of Project period - 4Q 2009 for case holding analysis (Table 6). Three treatment outcome parameters - cure rate, death rate and default rate were used to assess case holding among the low performing Districts. Two districts during 4Q 2007 had achieved a cure rate > 85%; an additional 3 districts achieved cure rates above 85% by the end of 4Q 2009 (totally 5 districts > 85% cure rate). However the number of districts with death rate (> 5%) increased from 5 in 4Q 2007 to 7 during 4Q 2009. The number of districts with default rate (> 5%) remained stable with 13 districts in the comparative period. The cure rate at state level observed a marginal increase from 81.6% to 82.3%, while the death rate and default rate strikingly remained comparable at 5.6% and 6.2% accordingly. Again any substantial impact of improved treatment outcomes as a result of ACSM specific targeted intervention approaches can only be pragmatically measured over a longer duration (3-5 year intervention).

**Table 6:** Treatment outcomes among low performing Districts, Odisha State 2007-2009

		4th Quarter 2007			4th Quarter 2009		
	District	Cure rate	Death rate	Default rate	Cure rate	Death rate	Default rate
1	Bolangir	83.2%	3.5%	6.9%	87.5%	3.7%	5.1%
2	Baleshwar	83.1%	7.4%	7.4%	80.2%	3.4%	9.6%
3	Bargarh	81.4%	7.1%	7.7%	83.0%	5.8%	5.8%
4	Bhadrak	85.7%	3.9%	6.5%	79.3%	4.9%	3.7%
5	Bhubaneswar	82.5%	4.8%	9.5%	88.7%	2.8%	7.0%
6	Cuttack	69.2%	8.1%	6.4%	86.1%	4.9%	4.9%
7	Debagarh	82.1%	2.6%	15.4%	80.6%	5.6%	11.1%
8	Ganjam	80.0%	5.4%	7.1%	76.1%	5.3%	8.0%
9	Kalahandi	64.0%	6.5%	12.4%	74.2%	5.5%	9.4%
10	Khordha	90.3%	4.3%	5.4%	86.2%	3.2%	9.6%
11	Koraput	80.7%	8.5%	3.4%	80.8%	8.5%	6.5%
12	Nabarangapur	83.5%	5.5%	6.3%	79.7%	5.9%	7.6%
13	Nayagarh	63.7%	3.2%	6.5%	58.2%	4.5%	12.7%
14	Puri	80.9%	2.6%	4.3%	78.6%	2.0%	6.0%
15	Rayagada	78.4%	5.4%	9.5%	87.5%	4.0%	7.7%
	**ODISHA STATE**	**81.6%**	**5.6%**	**6.2%**	**82.3%**	**5.6%**	**6.2%**

### Cost per reach

An attempt was also made to identify cost per reach (CPR) of the project interventions among a variety of social mobilisation activities implemented during the Project period (Table 7). These included reaching out to Women's SHG members, local self-governing institutions both at sub-district and village levels, slum community members, Village health sanitation committee members, traditional healers and special population groups.

**Table 7:** Cost per reach using multiplier

	Activity	**Nos**.	Participants reached	Unit cost (INR)	Total cost (INR)	Cost per reach (INR)	Cost per reach using multiplier (USD)	Level of influence - Multiplier	Total People reached using multiplier	Cost per reach using multiplier (INR)
1	SHG member sensitisation	4305	148326	500	2152500	14.51	**0.32**	10	1483260	1.45
2	Slum community sensitisation	560	12706	500	280000	22.04	**0.48**	10	127060	2.20
3	Special popln (fisher folk & stone quarry)	356	12070	500	178000	14.75	**0.32**	10	120700	1.47
4	PRI members sensitisation (at Village level)	485	9383	500	242500	25.84	**0.57**	100	938300	0.26
5	PRI members sensitisation at block level (Sub-district level)	7	435	1000	7000	16.09	**0.35**	500	217500	0.03
6	GKS member sensitisation	993	12528	400	397200	31.70	**0.70**	15	187920	2.11
7	Traditional healer sensitisation	100	2689	1500	150000	55.78	**1.23**	100	268900	0.56
8	Kalyani club sensitisation	8	297	500	4000	13.47	**0.29**	10	2970	1.35

* Multiplier- Number of people potentially influenced by sensitized participant over a period of 1 year (USD 1 = INR 45)

Table 7 data analysis suggests the average cost per person reached among the SHG members remains lowest at INR 14.51 (USD 0.32 cents), while the CPR for traditional healers was higher at INR 55.78 (USD 1.23). Interventions involving Self Help Groups, and local self-governing institutions were implemented across the state, whereas traditional healer interventions and village health sanitation committee meetings were more selectively implemented through targeted intervention approach in selected districts. While most social mobilisation activities implemented through the IFNGO's reduced coordination costs and had a lower CPR, project related coordination costs and consequently CPR were higher for interventions such as traditional healer interventions in tribal districts, where there had been challenges identifying capable IFNGO's.

In line with the theoretical foundations of diffusion theory [21,22], and the potential multiplier effect [23] many of the participants sensitised may have been perceived as 'opinion leaders' for TB and as such, may have further influenced other members of their communities. Discussions with the community, IFNGO's and Project coordinating staff, indicated that SHG members, who advocated for TB control with their constituents had the potential to influence at least an additional 10 people during the one year span of the Project. PRI groups that covered an average of 5000 population at village/Gram Panchayati (GP) level and 100,000 population at block level (sub-district) had the potential to sensitize at least 100 community members during the span of 1 year. The cost per reach for ACSM interventions can be seen to be dramatically reduced when this conservative multiplier is used with Table 7 illustrating the cost per person reached taking in to consideration this multiplier effect of health communication. Finally, a review of project plans and financial norms revealed that other than project specific IEC materials which were produced in adequate numbers for project activities, there was an RNTCP IEC budget allocation provided both at state and district level. However funds for these IEC activities, especially at district level, were found to be mostly underutilized.

## Discussion

The evaluation of the project indicated that the expanded use of ACSM activities including training, capacity building, adequate financial resources and specific targeted interventions in low-performing districts had a number of strengths. Key achievements of the Project were that it was found to have bridged the pre-existing gaps between the health system and the community, through the provision of support and coordination of efforts between key stakeholders-general health services, NGOs and the community. Findings showed that the ACSM interventions engaged a broad number of stakeholders, many of whom were previously not involved in TB control. Sensitisation activities were found to have also generally improved IPC skills, community and personal confidence, and increased community involvement and social mobilisation in TB control activities. This has led to greater community ownership of TB control, and extended networks of support. This should invariably lead to improved TB control services, positive attitudes and behavioural changes in the community, and ultimately, good clinical outcomes.

The general population approaches were shown to have provided support to a broad range of districts to further build capacity and re-energize the programme within community settings. The utilization of targeted interventions focused limited human and financial resources to those districts in most need of support, which was in line with the State ACSM Strategy recommendations. The strategic use of tailored messages to address specific TB problems in these low performing areas has also led to more positive behavioural outcomes specifically related to the TB challenges encountered in these areas. This has also improved efficiencies in service delivery in these resource constrained settings.

Nine specific recommendations for donors and stakeholders emanated from the evaluation including the following: continue targeted approaches to the lowest performing areas in alignment with the state ACSM strategy, continue tailoring of messages to address specific problems including case detection, default, patient and treatment delay, continue to build cooperative partnerships with all potential stakeholders to instill community ownership of TB and engage community groups for ACSM and other programme activities, continue engaging traditional/informal providers for referrals and involve them as DOT providers in tribal areas, build communication programming capacity and engage private sector partners in ACSM, empower TB patients and vulnerable groups, design innovative monitoring mechanisms to assess specific interventions and problem issues (i.e. case control studies), conduct succession planning to ensure future sustainability, maintain gains achieved to date, and momentum of Project in problem districts, and last, develop public health priorities for ACSM to optimize delivery of all state health priorities.

### Limitations

A number of project research limitations were identified during the evaluation. Limitations relate to participant's actual exposure to training and sensitisation interventions and the time lag prior to the rapid assessment. This may have reduced opportunities for recall of some of the interventions. This also highlights the challenges in establishing attribution for ACSM which was only one component of a comprehensive TB control programme.

Additionally, it was noted that limitations related to the Project also impacted on the outcomes achieved which were identified through the review. The main reason for this related to the issue of sustainability due to the fact that the Project implementation period (21 months in-field activities during the 30 month project timeline) was insufficient to assess behavioural impact in relation to treatment outcome objectives-case detection, case holding, patient and treatment delay. From a Project perspective the IFNGO concept was also identified through the review as an innovative approach. However, additional follow-up would be required and extended financial support to fully assess and institutionalize this model.

Finally, project limitations in terms of the lack of organisational set-up at the field level and inadequate funding for the ACSM Project activities (mass media), may have limited the impact of the interventions and subsequent findings. In particular, the lack of any mass media materials that was not within the scope of the Project, other than community based IEC materials was noted. Given the important supporting role of mass media to set health agendas and support community based activities [24,25], this was seen as a shortcoming which should be addressed in future programmes, thus ensuring future evaluations the potential synergistic effects that multilevel ACSM programming could achieve.

## Conclusion

The use of rapid assessment and response (RAR), and other complementary qualitative and quantitative evaluation approaches as a means for undertaking a comprehensive review of the impact of TB ACSM activities in Odisha State is found to be a fruitful approach. The survey method achieved in a relatively short time period, a comprehensive understanding of the characteristics of the TB related health problems in Odisha, risk behaviours, and social consequences. The rapid assessment identified the positive impact that a comprehensive and well planned range of ACSM activities can achieve in enhancing TB KAB as well as engaging and mobilising specific community groups to build greater community efficacy to combat TB. Most importantly, the RAR methodology and supporting empirical data analysis was found to provide a more comprehensive range of performance parameters than singularly qualitative or quantitative assessment approaches. This provided for a broader range and higher quality key recommendations for donor and Odisha stakeholders consideration, a number of which could be implemented to achieve even greater cost efficiencies and continuous improvement in the ACSM program in years to come. Despite these encouraging findings, important to note is the fact that any substantial impact in terms of improved programme related case finding and treatment outcomes as a result of ACSM specific approaches can only be pragmatically measured over a longer duration, 3-5 year intervention period.

## List of abbreviations

ACSM: Advocacy, Communication and Social Mobilisation; ARTI: Annual Risk of TB Infection; ASHA: Accredited Social Health Activist; AWW: Anganwadi Worker; AYUSH: Ayurveda, Yoga, Unani, Siddha and Homoeopathy; BEE: Block Extension Educator; BPO: Block Programme Officer; CBO: Community Based Organisation; CDMO: Chief District Medical Officer; CF: Communication facilitator; CTD: Central Tuberculosis Division; DANIDA: Danish International Development Agency; DMEIO: District Mass Education and Information Officer; DOHS: Directorate of Health Services; DOT: Directly Observed Treatment (TB); DOTS: Directly Observed Therapy - Short course; DPM: District Programme Manager; DTO: District TB Officer; ESI: Employees State Insurance; GFATM: The Global Fund to fight AIDS, Tuberculosis and Malaria; GHS: General Health Staff; GKS: Gaon Kalyan Samitis (Village Health Sanitation Committee); GOI: Government of India; GP: Gram Panchayati (Village self-government bodies); HIV/AIDS: Human Immunodeficiency Virus/Acquired Immune Deficiency Syndrome; IEC: Information, Education and Communication; IFNGO: Interface Non-Governmental Organisation; IMA: Indian Medical Association; IPC: Interpersonal Communication; KAB: Knowledge, Attitudes and Behaviours; KAP: Knowledge, Attitudes and Practices; LT: Laboratory Technician; M&E: Monitoring and Evaluation; MDR-TB: Multidrug-Resistant Tuberculosis; MO: Medical Officer; MO-DMC: Medical Officer - District Medical Centre (MO-DMC); MO-IC: Medical Officer In-Charge; MOU: Memorandum of Understanding; MPHS: Multipurpose Health Supervisor; MPHW: Multipurpose Health Worker; NGO: Non-Governmental Organisation; NRHM: National Rural Health Mission; PHC: Primary Health Center; PRI: Panchayati Raj Institutions (local self-government bodies); RAR: Rapid Assessment and Response; RNTCP: Revised National Tuberculosis Control Programme; SHG: Women's Self Help Group; SSI: Semi-Structured Interview; STS: Senior Treatment Supervisor; TB: Tuberculosis; The Union: International Union Against Tuberculosis and Lung Disease; WHO: World Health Organisation.

## Competing interests

The authors declare that they have no competing interests.

## Authors' contributions

VVK and TT conceived and designed the assessment methods and instruments, participated in the evaluation and drafted the manuscript; manuscript review and edits by NW and SS. NW, SS and LSC contributed to interpretation and writing. All authors read and approved the final manuscript.

## Pre-publication history

The pre-publication history for this paper can be accessed here:

http://www.biomedcentral.com/1471-2458/11/463/prepub
